# Why People Play: Artificial Lives Acquiring Play Instinct to Stabilize Productivity

**DOI:** 10.1155/2012/197262

**Published:** 2012-12-03

**Authors:** Shinichi Tamura, Shoji Inabayashi, Waichi Hayakawa, Takahiro Yokouchi, Hiroshi Mitsumoto, Hisashi Taketani

**Affiliations:** ^1^NBL Technovator Co., Ltd., 631 Shindachimakino, Sennan 590-0522, Japan; ^2^Image Processing Solutions Deptartment, Pacific Systems Corporation, 8-4-19 Tajima, Sakura-Ku, Saitama City 338-0837, Japan; ^3^Osaka Electro-Communication University, 18-1 Hatsucho, Osaka, Neyagawa 572-8530, Japan; ^4^Tsuyama National College of Technology, 624-1 Numa, Okayama, Ttsuyama 708-8509, Japan

## Abstract

We propose a model to generate a group of artificial lives capable of coping with various environments which is equivalent to a set of requested task, and likely to show that the plays or hobbies are necessary for the group of individuals to maintain the coping capability with various changes of the environment as a whole. This may be an another side of saying that the wide variety of the abilities in the group is necessary, and if the variety in a species decreased, its species will be extinguished. Thus, we show some simulation results, for example, in the world where more variety of abilities are requested in the plays, performance of the whole world becomes stable and improved in spite of being calculated only from job tasks, and can avoid the risk of extinction of the species. This is the good effect of the play.

## 1. Introduction

Life is a highly rationalized organization. There often exists hidden rationality even in an irrational action at a glance. It is often said that in the human organization the top a-third people work hard and draw the organization, but the bottom a-third are lazy and work as brakes for the development of the organization. However, if a new organization is made with only this bottom a-third, also new top a-third people are born from them as excellent constituents and draw the organization. Generally speaking, each individual has his or her own peculiar and unique abilities, and he or she exhibits the hidden ability when it is requested. Human society is composed of divided works and specialized individuals with various explicit and implicit abilities. There are some work in the literature dealing with artificial brain and mind [1–4]. However, behavior of various abilities embedded into mankind has not been dealt with.

**Figure 1:**
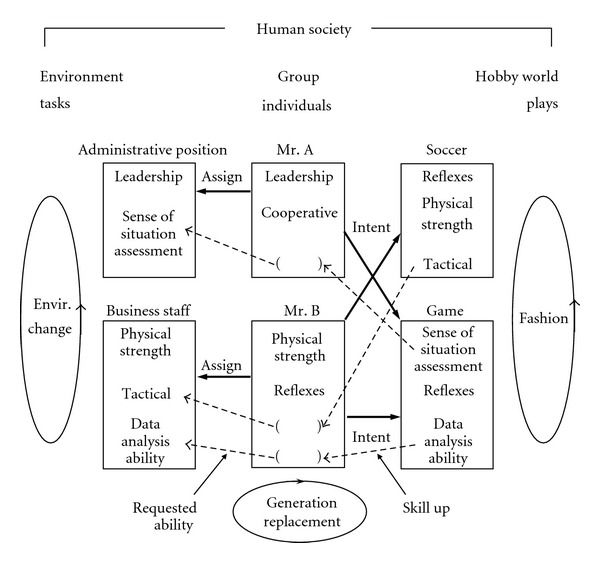
Gene world model with interaction to external environment. () shows lacking ability.

We assume that human society is composed of individuals, business positions (tasks), and hobby world with various plays. Human society has an interaction with the outer world (environment) which requests to the human society various tasks. For example, if the density of CO_2_ increases, a task to decrease or suppress it is requested to the human society from the environment. Further, if the internet becomes spread over the human society, a task to utilize it is requested from the environment although it is a product of human society and somehow artificial. Such environment varies from time to time. To cope with such environmental changes, human society must make their work or business system change by assigning to the tasks (positions) individuals with adequate abilities or reorganize them. However, each individual is not expected to have enough abilities which are requested in the tasks. In our model shown in Figure 1, lacking abilities are trained and their skill is increased in the plays.

The play is often referred to as an opposite action to the diligence. However, like the play at housekeeping of the children, if we consider it as the training occasion of the social roles, the meaning of the play becomes largely different. Competitive playing sports may also be regarded as a hidden side of hot war to avoid destructive results by applying rules. The human society is requested to continuously cope with variously changing environment with its variety of abilities. At ordinary times most part of such abilities are hidden as they are, and a part of it is revealed as the play and training. 

In this paper, using genetic algorithm we generate a group of artificial lives which are capable of coping with various environments which is a set of requested tasks. Under such situation, we like to show that the plays or hobbies are automatically generated as a necessary instinct or they are a natural result of evolution of the group of individuals in order to maintain the coping capability with various changes of the environment as a whole. This may be another side of that the wide variety of the abilities in the group is necessary and if the variety in a species decreased, its species will be extinguished. 

This paper shows an attempt to generate an artificial mind which has two aspects of “diligent to his job” and “apt to run to pleasure.” We show that the latter does not have a negative meaning but also has a positive meaning to cope with variously changing environment as a group and stabilize the world productivity. 

## 2. Gene World Model

### 2.1. A Set of All Kinds of Human Abilities

In this paper we assume that human abilities are countable such as

(1) $$\begin{array}{c} a_{1}=\left[\text{swift-of-foot}\right],\\ a_{2}=\left[\text{patient}\right],\\ a_{3}=\left[\text{skillful-in-calculation}\right],\\ a_{4}=\left[\text{fair-spoken}\right],\\ a_{5}=\left[\text{rich-in-leadership}\right],\\ a_{6}=\left[\text{rich-in-curiosity}\right],\\ a_{7}=\left[\text{rich-in-competitive-spirit}\right],\\ a_{8}=\left[\text{aggressive}\right],\\ a_{9}=\left[\text{interest-in-fishing}\right]. \end{array}$$

The set of such abilities is defined as

(2) $$\begin{array}{c} A\;=\left\{a_{1},a_{2},\ldots ,a_{NA}\right\},\;\;\left(\text{Ability}\;\text{set}\right). \end{array}$$

Null element may be included in *A* to make the following expressions simple, by which the sizes of some of the following sets may be made fixed by filling with it.

### 2.2. Environment

An environment (outer world) *Et* at time *t* is expressed as a set

(3) $$\begin{array}{c} Et=\left\{T_{t1},\ldots ,T_{tnt}\right\};\;t=1,2,\ldots ,\;\;\left(\text{Environment}\right) \end{array}$$

of requested tasks in the environment, where tasks are, for example,

(4) $$\begin{array}{c} T_{\text{old-times}\;1}\;=\left[\text{protect-from-wild-animals}\right],\\ T_{\text{recent-ages}\;1}\;=\left[\text{publish-book}\right],\\ T_{\text{recent-ages}\;2}\;=\left[\text{open-a-store}\right],\\ T_{\text{recent-ages}\;3}=\left[\text{negotiate-with-other-company}\right],\\ T_{\text{today}\;1\;}=\left[\text{set-computer-business-up}\right]. \end{array}$$

The environment changes from time to time.

### 2.3. Task

Each task *Ti* is expressed by a set of requested abilities to accomplish it as

(5) $$\begin{array}{c} Ti=\left\{a_{i1},a_{i2},\ldots ,a_{ini}\right\};\;i=1,\ldots ,N_{T},\;\;\left(\text{Task}\right). \end{array}$$

For example,

(6) T1(=task  of  [protect-from-wild-animals])  ={quick-motion,cool-mind,good  ear,…}.T2(=task  of[political-action])  ={fair-spoken,planning-ability,…}.

## 3. Play

On the other hand, there is a set *Pt* of all plays (hobbies) including self-development and volunteer activities to which people begin or join voluntarily at time *t*. That is,

(7) $$\begin{array}{c} Pt=\left\{R_{t1},\ldots ,R_{tmt}\right\};\;t=1,2,\ldots ,\left(\text{Play}\;\text{set}\right). \end{array}$$

For example,

(8) Pt={soccer,go-game,marathon, reading,travel,fishing,volunteer-activity,…};                      t=today.

Each play also requests a set of abilities:

(9) $$\begin{array}{c} Ri=\left\{a_{i1},a_{i2},\ldots ,a_{imi}\right\};\;\;i=1,\ldots ,N_{R},\;\;\left(\text{Play}\right). \end{array}$$

For example,

(10) R1(=play  of  [fishing])  ={interest-in-fishing,driving  car,patient,…},

(11) R2(=play  of  [go-game])  ={interest-in-go-game,pattern  recognition,   spatial  reasoning,tactical  sense,…}.

Abilities of [interest-in-*] are always necessary to begin the play of *. The set *Pt* of plays may also change from time to time according to the fashion. Only the difference between the task and the play is that the task is passively given or controlled by the environment, and the play is sought voluntarily or begun by urge and hard to stop.

### 3.1. Individual

Each individual *Ik* has his abilities and their skills:

(12) Ik={ak1,…,aknI;  sk1,…,sknI;  ck};k=1,2,…,NI,  (Individual),

where *s* is a corresponding skill and *p* is a rate of spending time for the play where the hidden as well as the explicit abilities are trained. The set of individuals is generated and evolved by GA algorithm. Skills are raised by the experience in the job task or in the play but not inherited. The abilities and the ratio *c* are inherited across the generations. When one has more than two hobbies, time ratio *c* may be divided among them, or only the most fitted one is selected as shown in Section 3.4 and applied in the experiment.

#### 3.1.1. Formulation

A simple example of the world model is shown in Figure 1. The outside environment is always changing. It is the origin of the evolution. In this section, some basic formulas of the simulation are given.

### 3.2. Skill

We assume here the skill is given by

(13) $$\begin{array}{c} s_{ki}=0.5+\sum_{t}\left[\alpha_{ki}^{T}\times \left(1-c_{k}\right)+\alpha_{ki}^{P}\times c_{k}\right]\;\;\left(\text{Skill}\right) \end{array}$$

which is roughly proportional to the experienced time (years, or unit years); the ability *a*_*ki*_ was used in the task or the play. *c*_*k*_ and 1-*c*_*k*_ are rates of time used for the play and the task, respectively. For simplified example, in case of one working full time in weekdays and spending only for play full time in weekend, *c*_*k*_ = 2/7 and 1-*c*_*k*_ = 5/7 by neglecting another time, for example, housekeeping. The value 0.5 is an initial skill when the individual has no experience. This means that one can do even unexperienced task to some extent using other means. *α*_*ki*_^*T*^ takes 1 when the ability *a*_*ki*_ is used in the task and 0 when not used. *α*_*ki*_^*P*^ is that of the play. The value of skill will be upper limited by their life-span automatically.

#### 3.2.1. Assignment

When the environment Et is given, an individual most fitted to the task (see Section 3.3) among randomly selected unassigned *Ne* (e.g., five) individuals is assigned to the task. Individuals not assigned to any task are called “window-side-folks” and direct his energy to the play with the time rate of

(14) $$\begin{array}{c} \min\left[1,b\times c_{k}\right];\;b\geq 1\;\left(\text{e}.\text{g}.,\;\;b=2\right). \end{array}$$

Then the skills used implicitly as well as explicitly in the play will be increased as much.

### 3.3. Fitness to Task

The fitness of the individual *Ik* =  {*a*_*k*1_,…, *a*_*k* *n*I_; *s*_*k*1_,…, *s*_*k* *nI*_; *c*_*k*_} to the task *Ti* = {*a*_*i*1_, *a*_*i*2_,…, *a*_*ini*_} is given by sum of the skills used in the task as

(15) $$\begin{array}{c} f_{ik}=\left(1-c_{k}\right)\times \sum_{j=1}^{n_{i}}s_{kj}\;\left(\text{fitness}\right) \end{array}$$

such that *a*_*ij*_ = *a*_*kl*_ for some *l*  in {1,2,…, *n*_*I*_}. 

If there are two same abilities in an individual, its skill is counted twice, and so on. That is, for example, if one has three genes of the same ability of [swift-of-foot], he may have three times of the skill of the [swift-of-foot]. On the other hand, if he has no such ability, *s*_*kl*_ is counted as 0.5 (min). Though this process may be more nonlinear, for the ease of modeling it was made linear.

### 3.4. Fitness to Play

Also for the plays we can consider the same fitness, and each individual selects the most fitted Ri among the set of plays specified by ability of [interest-in-*] and practicing it. If one has no such ability, he will spend the leisure time with rate *c* idly; that is, his abilities are not trained in this time.

### 3.5. Performance of the Whole World

Performance of the whole world is given by

(16) Q=∑i=1NTfi∗  (performance),

where  *f*_i_* is *f*_*ik*_ of an individual *Ik* who is practically assigned to the task *Ti*. Note that hobbies are not evaluated at all in this model.

### 3.6. Evolution of Individuals

Genetic algorithm [5, 6] is employed to make the evolution of the set of individuals. At every year (or unit year), some percentages of the individuals with fitness in order from the lowest or randomly to some extent are selected and erased. Then, those with the highest fitness are multiplied.

### 3.7. Genotype

In order to simulate the artificial humans we must map the model to genotype. It may be more simple to represent the individual (*Ik* *'* = {*a*_*k*1_,…, *a*_*knI*_; *c*_*k*_}) by a sequence of binary numbers with (log⁡_2_⁡*N*_*A*_ × *n*_*I*_ + bit  length  of  *c*_*k*_) bits. Since skills are not inherited, {*s*_*k*1_,…, *s*_*knI*_} is not included in the *Ik* *'* different from *Ik*.

The environment is given from the outside to the group of individuals. It requests the tasks. On the other hand, the plays are generated as a result of the evolved genes. To do so, some specific binary sequences on the gene should be interpreted as [interest-in-*] with which he or she is represented as being fond of that kind of play and begin to do it. The set of trainable abilities in each play is given also from the outside.

## 4. Simulation and Experimental Results

We have developed a simulation software. 

### 4.1. Parameters

 The initial parameters of the simulation are *N*_*A*_ = 128 (number of kinds of ability), *N*_*I*_ = 120 (number of populations), *N*_*T*_ = 100 (number of job tasks; 20 are unemployed), *n*_*i*_ = 16 (number of abilities required in each job tasks), *n*_*i*_ = 16 (number of abilities each person has), *N*_*R*_ = 64 (number of kinds of play), *m*_*i*_ = 0–64 (number of abilities required in each play), *b* = 2, *c*_*k*_  = [7bits], and the rate of mutation is 0.05. Zone of the ability code number more than 64 (i.e., 65–128) is allocated to the abilities of {interest-in-*}. Initial genes are set randomly. 

### 4.2. Stepwise Environmental Change

**Figure 2:**
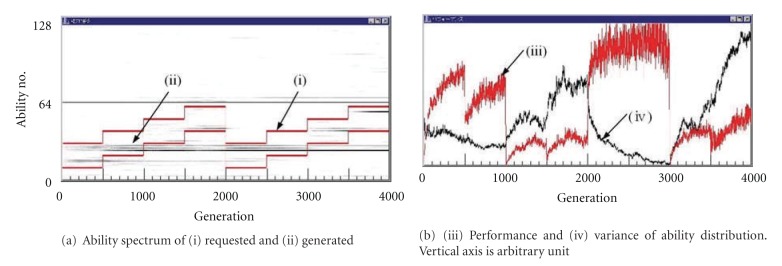
Typical behavior of the world with 64 kinds of less complicated plays each of which requires no ability.

Figure 2 shows a typical behavior of the world according to the generation lapse with 64 kinds of less complicated plays each of which requires no ability. In Figure 2(a), the vertical axis represents the code number of the ability which is expressed by seven bits in the gene. The environment requests abilities between two lines (i) with band width 20 and changing stepwise according to the generation. Requested 16 (=*n*_*i*_) abilities for each task are selected from this band of 20 randomly. Then, various abilities (ii) in the group of the artificial humans become to appear. That is, (ii) represents a spectrum of ability set embedded in the artificial mankind and changing along the time. When the generated ability distribution as a result of the evolution matches well to the requested abilities, the performance (iii) of the world becomes high. When the environment change effects largely in the world, the performance of the world becomes almost zero and has a risk of extinction of the species as in the 1000th, 1500th, and 3000th generation. On the other hand, the change at the 2000th generation will be likened to the Industrial Revolution, where the performance of the world is raised up in spite of the sudden change of the environment (requested tasks) since the abilities in artificial mankind fit well to it. The curve (iv) shows the variance of the ability distribution (ii). We can see that when the ability distribution (ii) matches well to the environment (i) with small variance, the performance becomes very high as in between the 2000th and 3000th generations. Abilities between no 64 and no 128 that correspond to {interested-in-*} in Figure 2(a) are dispersed and thin, since the abilities of {interested-in-*} do not contribute to the performance.

**Figure 3:**
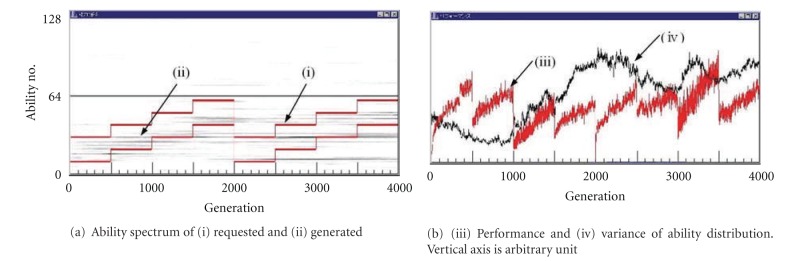
Typical behavior of the world with 64 kinds of highly complicated plays each of which requires 64 abilities.

Figure 3 shows a typical behavior of the world with 64 kinds of highly complicated plays each of which requires 64 abilities. We can see that the performance of the world is fairly steady compared with Figure 2. The variance curve is also steady than Figure 2.

We can see that in the world where more varieties of abilities are requested in the plays, or various kinds of plays are possible and popular as in the advanced countries, the performance of the whole world in spite of calculated only from tasks becomes more stable by the continuous skill up in the plays. This is the effect of the play. 

In the first and the second simulations of Figures 2 and 3, the abilities of {interested-in-*} were only for plays. We have made the third simulation where we have defined the ability of {interested-in-task*}. The ability of {interested-in-task*} is not always necessary to begin the task*. When individuals are assigned to tasks, the ability of {interested-in-task*} is used as an extra ability. Then, the performance is higher than those who don't have it as much as its skill. Thus, his skill is raised up more since he has more chance to be employed or promoted more, and it makes more chance to get good job, and so on. 

**Figure 4:**
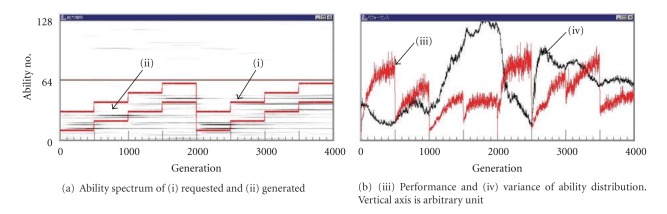
Behavior of the world with 64 kinds of less complicated plays requiring no ability as Figure 2 but abilities of interest-in-task added.

Besides the abilities with code numbers 65–128 are allotted to plays one-to-one, each task is allotted randomly to one of the code numbers of 65–128. That is, each the code number between 65 and 128 is allotted to one play and 0, 1, or 2,… tasks. An experimental result is shown in Figure 4, where the performance is more stable than Figure 2 and the risk of the extinction seems decreased. This may be because individuals having interest-in-task ability have more chance to fall in a good circulation of being selected and polish Their abilities. However, note that the stability is still lower than the world with the complicated plays of Figure 3 in this case. 

 We can say that there is a discrimination between the tasks as the duty one and the voluntary one with interest. This is the effect of the interest in this task. We can see that the improvement of the ability by which they performed the task voluntarily contributes to the variance of the ability. We can find that there are common parts between the {interested-in-task*} and the voluntary play, for example, the baseball is played as a profession and as a play (hobby). So we may be able to say this is the effect of the play.

### 4.3. Random Environmental Change

**Figure 5:**
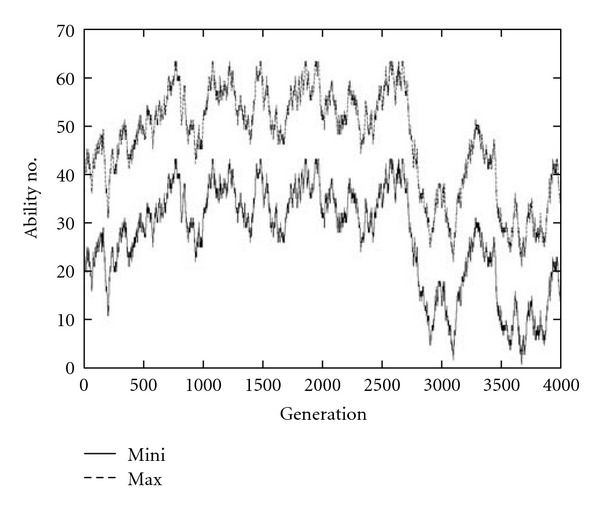
Random environmental change. Range of 20 between upper and lower curves is the requested abilities in each generation.

In order to see the difference by complexity of the plays from statistical point of view, we performed 100 trials of the simulation under the random environmental change shown in Figure 5. 

**Figure 6:**
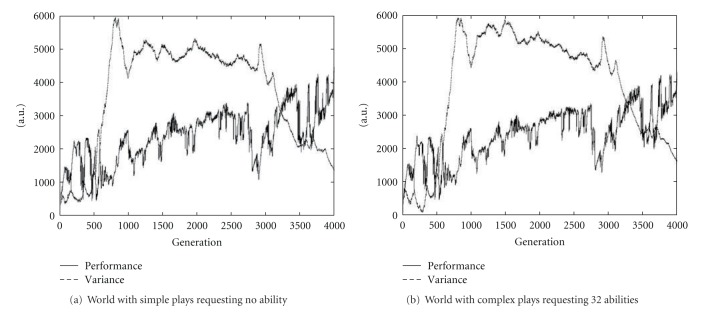
Average of 100 trials of the world. Lower dark curve is the performance and upper thinner curve is the variance of the gene spectrum.

 Results are shown in Figure 6. Comparing performance and variance between worlds with different complexity of the play for the same environmental change, there are not so much differences at a glance in (a) and (b) of Figure 2. Therefore, the mean differences are calculated as Table 1. We can see that when the plays request more number of abilities, performance of the world becomes higher, and spectrum of genes within the world becomes more spread compared with the world with only simple plays. That is, there become people that have more wide range of abilities and talents. Consequently, in the play-rich world, though productivity is not always higher, for sudden environmental changes drop of the productivity is small and the productivity after the drop is kept higher than in the simple play world as seen at around 2700 generation and 3400 generation in Figure 6. Though these differences do not look so large, they are accumulated and make a large difference. 

Note that in the simulation, abilities are fixed beforehand. In real situations, however, they are newly generated to adapt more to the varying environment. Introducing this mechanism to the simulation, it will make the simulation more realistic. For example, in both worlds in Figure 6, we see that the variances after 3000 generation are decreasing though the performances are still raising and they show the limit of evolution by being too much adapted to the environment. These limits will be removed by the above improvement. 

## 5. Discussion

Generally speaking, the simulation of GA is not always stable, but sometimes it happens that even when the performance is growing up steadily the evolution becomes bad suddenly. This is partly because of the limited number of individuals in the above simulation.

Though we have not tried fully yet, it is expected that a group of individuals with wide variety of abilities as a whole is generated by the effect of varying environment. In other words, required wide variety of abilities to cope with variously changing environment are embedded distributively in the group. As a result each individual comes to have his own personality and uniqueness. Individuals constitute the world (group or independent country) by sharing tasks required in the present environment. However, the whole abilities embedded in the group distributively are not only the ones required in the present environment but also for its possible changes. Some parts of such abilities embedded into an individual will appear as plays or hobbies. Thus, in spite of only evaluating the fitness of abilities of the individual to the changing environment, not only the variety of the abilities for the tasks but also the variety of the abilities for plays come to appear. As a result, three types of individuals will be generated. The first type of the individual is fitted to his present task, and this type of individual is happy only in his job. The second one is happy in his play rather than in his job. The third one is their intermediate type. Essentially, it may be nonsense to discriminate the abilities for the job tasks and for the plays, although in this paper we discriminated them for convenience in the simulation.

The present environment requests not the whole abilities of the group of humans but only the part. The unrequested abilities will appear as the play in the hobby world and prepare the next chance to be requested from the environment by being trained in the play.

Though the paper deals with a world of mankind, the frame of the research will also be applicable to various worlds with struggle for existence, such as animal world and business world.

## 6. Conclusion

In this paper, we have proposed a gene world model of a group of artificial humans capable of coping with various environments, where individuals with mind of plays or hobbies are generated. We have shown in a simulation that in the world, where more variety of abilities are requested in the plays, the performance of the whole worlds in spite of calculated only from tasks becomes stable. This is the effect of the play. Thus, we may be able to say that we could confirm that the role of plays will be partly some kind of training for implicit or explicit abilities which will be useful for unexpected changes of the environment. It can be regarded as play expresses a margin of ability.

Further improvement along the following items will be necessary.

(1) The system behavior is rather dependent on how the environment changes in speed and range. Therefore, we must investigate what type of changes will generate what type of artificial lives.
(2) Structure of the model of the task is flat of only a personal job level. It might be needed to construct a task system with a hierarchical structure such as a company composed of individuals.
(3) It may be difficult in the real world to discriminate the job tasks as the duty one and the hobbies as the voluntary one with pleasure. It may be a way not discriminating [interested-in-play*] and [interested-in-task*].
(4) In the real world, evaluation of the world is made not only by the job tasks but by including the plays.
(5) “Variance” may not be adequate since it depends on how to allot the number to the ability. Therefore, entropy may be more adequate to express the diversity of the gene.
(6) Though in the simulation, abilities in the mankind are fixed beforehand in the real situation they are evolved to adapt more to the changing environment. Then, the artificial lives will evolve more drastically.

## Figures and Tables

**Table 1:** Improvement of means of performance and variance from simple play world. Values are arbitrary unit in Figure 6.

Abilities for play	Performance	Variance
0	—	—
8	18	39.26
16	35	38.57
32	14	41.61
